# Metabolic syndrome in systemic lupus erythematosus was closely related to body mass index, blood pressure, blood sugar, blood lipids, and arthritis

**DOI:** 10.12669/pjms.36.6.2093

**Published:** 2020

**Authors:** Lai-Run Jin, Meng-Jun Tao, Jun Zhou, Liang Xu, Qiang Li, Zhi Li, Hui Peng, Hui Yuan

**Affiliations:** 1 Lai-Run Jin, Office of Educational Administration, The First Affiliated Hospital of Wannan Medical College (Yijishan Hospital of Wannan Medical College), Wuhu 241001, China; 2 Meng-Jun Tao, School of Public Health, Wannan Medical College, Wuhu 241001, China; 3 Jun Zhou, School of Public Health, Wannan Medical College, Wuhu 241001, China; 4 Liang Xu, Department of Rheumatology, The First Affiliated Hospital of Wannan Medical College (Yijishan Hospital of Wannan Medical College), Wuhu 241001, China; 5 Qiang Li, Department of Medical Record, The First Affiliated Hospital of Wannan Medical College (Yijishan Hospital of Wannan Medical College), Wuhu 241001, China; 6 Zhi Li, Department of Rheumatology, The First Affiliated Hospital of Wannan Medical College (Yijishan Hospital of Wannan Medical College), Wuhu 241001, China; 7 Hui Peng, Administration Office of Hospital Admission and Discharge, The First Affiliated Hospital of Wannan Medical College (Yijishan Hospital of Wannan Medical College), Wuhu 241001, China; 8 Hui Yuan, School of Public Health, Wannan Medical College, Wuhu 241001, China

**Keywords:** Metabolic syndrome, Random forest algorithm, Relevant factors, Systemic lupus erythematosus

## Abstract

**Background and Objective:**

Prevention and control of metabolic syndrome is the key to improving the development of systemic lupus erythematosus. The aim of this study was to analyze the relevant factors regarding metabolic syndrome (MS) in systemic lupus erythematosus (SLE).

**Methods:**

A total number of 1238 SLE patients in Yijishan Hospital of Wannan Medical College, Anhui province, from February 2012 to July 2017, were analyzed retrospectively. SLE patients with MS were grouped to group SLE-MS, the others without MS was grouped to group SLE-nMS. The two groups were compared with respect to general characteristics, clinical signs, and laboratory parameters. Random forest approach and multivariate logistic regression were conducted to analyze the related factors regarding MS in SLE.

**Results:**

The constituent ratio of metabolic syndrome was 27.14% (336/1238). More SLE patients with MS presented with more farmers, more married people, lower education level, and more lupus nephritis, proteinuria, oral ulcers, tubular urine, hematuria than SLE patients without MS (*P*<0.05). Moreover, eighteen important variables, whose average importance scores were highest and whose error rates were lowest, were selected by random forest method. Data from multivariate logistic regression showed that MS in SLE was related with BMI, diastolic blood pressure, systolic blood pressure, fasting blood glucose, arthritis, urea, triglycerides, high-density lipoprotein, and white blood cells.

**Conclusion:**

MS in SLE was closely related to BMI, blood pressure, blood sugar, blood lipids, arthritis, white blood cells, and urea. Targeted prevention and conclusion measures for the risk factors should be taken as early as possible.

## INTRODUCTION

Systemic lupus erythematosus (SLE) is an autoimmune-mediated diffuse connective tissue disease characterized by immune inflammation. The incidence of SLE in the world is about (7.4~159.4)/100,000, which seriously endangers the physical and mental health of residents and becomes an important global public health problem.^1^ The majority of SLE patients were women, and the incidence of myocardial infarction, congestive heart failure, and stroke in SLE women increased by 8.5 times, 13.2 times, and 10.1 times, respectively, and SLE patients developed menopause 3-4 years earlier than ordinary women.^2^ The incidence of hypertension, diabetes, elevated very low-density lipoprotein, elevated triglycerides and atherosclerosis in SLE patients is significantly higher than that in the general population, and the absolute risk of coronary heart disease in SLE exceeds that in Type-2 diabetes.^3^-^5^ These studies have shown that the risk of cardiovascular disease (CVD) in SLE patients is significantly higher than in the general population.

The late cause of death in patients with SLE is primarily CVD. The increase in CVD risk in SLE is caused by multiple factors. Selzer et al. selected 214 women with SLE who had no clinical manifestations of cardiovascular disease and found that carotid plaque formation and hypertension were associated with a decrease in high-density lipoprotein.^6^ Hyperlipidemia and hyperglycemia are independent risk factors for intimal thickening, and hardening of the aorta is also associated with hypertension. Studies have found that serum total cholesterol (TC) greater than 5.2mmol / L can predict the occurrence of coronary heart disease (CHD) in SLE patients, 24% of patients with elevated TC levels have CHD, and only 3% of patients with normal TC levels developed CHD.^7^ The role of these traditional risk factors in the development of atherosclerosis in SLE patients cannot be ignored, and metabolic syndrome (MS) is just a collection of risk factors, that is, central obesity and insulin resistance as the central link, with hypertension, a group of metabolic disorders with hypertriglyceridemia, impaired glucose tolerance, or type 2 diabetes as the main clinical manifestations.^8^ MS is an independent risk factor for CVD in patients with SLE. So prevention and control of MS is the key to improving the development of SLE.^9^,^10^ This study analyzed the data of 1238 patients with SLE in order to explore the relevant factors of MS in SLE patients.

## METHODS

A total of 1238 SLE patients including 143 males and 1095 females, 336 with MS, 902 without MS, with an average age of 41.64±22.92 years old, were recruited from Affiliated Yijishan Hospital of Wannan Medical College. All patients were new-onset cases first diagnosed by hospital physicians. SLE was defined based on the classification criteria revised by the American College of Rheumatology (ACR) in 1997.^11^ Disease activity was assessed based on the Systemic Lupus Erythematosus Disease Activity Index 2000 (SLEDAI).^12^ MS was defined based on the diagnostic criteria revised by the Chinese diabetes society (CDS) in 2004.^13^ Three or more of the following four components are defined as MS:

- Overweight or obese, BMI ≥ 25kg / m 2.
- Hyperglycemia, fasting blood glucose > 6.1 mmol / L, or two hour postprandial blood glucose > 17.8 mmol / L, and / or have been diagnosed with diabetes and treated.
- Hypertension, systolic blood pressure ≥140 mmHg and / or diastolic blood pressure ≥ 90 mmHg, or have been diagnosed with hypertension and treated.
- Hypertriglyceridemia, triglyceride ≥ 1.7 mmol / L, and high-density lipoprotein cholesterol < 0.9 mmol / L for male, and < 1.0 mmol / L for female.

Exclusion criteria for all patients were as follows:

1. Patients with anemia.
2. Patients with malignancies.
3. Patients with pregnancy.
4. Patients with viral infectious diseases.
5. Patients with other autoimmune diseases.

General characteristics, clinical signs, and laboratory parameters were obtained through epidemiological interviews and hospital records. Patients were randomly selected from ligible cases in the ward of the hospital’s Rheumatology Department based on their hospitalization number and a random number table.

### Statistical analysis

Epidata 3.0 software was used to enter data, and SPSS 17.0 software was used for single factor analysis and multivariate analysis. The R 3.5.1 software RandomForest command package is used for dimensionality reduction and initial screening of variables. We use the random forest algorithm to sort the importance of variables, run the sliding window sequential forward feature selection (SWSFS) process,^14^ plot the error rate graph, and combine the variables with the lowest error rate into the binary logistic regression model. Numerical data conforming to normal distributions were presented as means± standard deviation (SD); those not normally distributed were presented as medians (interquartile range, IQR). Mann-Whitney rank sum tests or Two-tailed Student’s *t*-tests for independent samples were performed to estimate differences between groups for continuous variables. The Chi-squared test and Fisher’s exact test were used to assess differences in categorical variables among groups. A two-tailed *p* value <0.05 was taken to be statistically significant.

### Ethics approval and consent to participate

The study was approved by the Ethics Board of Wannan Medical College (Number: 2016032; date: December 15, 2015). All patients gave informed consent for publication in the research study and patient privacy was protected.

## RESULTS

Participants with MS accounted for 27.14% of SLE patients. The age (*P*=0.145) and gender (*P*=0.662) between the SLE-MS group and the SLE-nMS group were not statistically different. The proportion of farmers, married people and low education level in the SLE-MS group was statistically higher than that in the SLE-nMS group (*P*<0.05) (Table-I)

**Table-I:** Comparison of general information between SLE-MS group and SLE-nMS group [n(%)].

Variables	n	SLE-MS	SLE-nMS	χ^2^	P
***Gender***					
Male	143	41 (12.20)	102 (10.28)	0.192	0.662
Female	1095	295 (87.80)	800 (80.65)
***Age***					
11~	29	5 (1.49)	24 (2.42)	5.391	0.145
18~	560	166 (49.40)	394 (39.72)		
45~	319	88 (26.19)	231 (23.29)		
60~	103	21 (6.25)	82 (8.27)
***Occupation***					
^a^Farmer	272	85 (25.30)	187 (18.85)	16.185	0.003
^ac^Unemployed	552	167 (49.70)	385 (38.81)		
^bc^Worker	125	26 (7.74)	99 (9.98)		
^bc^Student	57	8 (2.38)	49 (4.94)		
^b^Other	232	50 (14.88)	182 (18.35)
***Marital status***					
^a^Married	1062	305 (90.77)	757 (83.92)	11.766	0.003
^bc^Unmarried	169	28 (8.33)	141 (15.63)		
^ac^Divorced or Widowed	7	3 (0.89)	4 (0.44)
***Educational level***					
^a^Elementary school and below	382	165 (49.11)	217 (24.06)	72.793	<0.001
^b^Middle school	625	130 (38.69)	495 (54.88)		
^b^College and above	231	41 (12.20)	190 (19.15)		

***Notes:*** Different superscript letters denote significant differences (P<0.05).

Significant results are highlighted in bold type.

The proportion of participants with lupus nephritis (LN), proteinuria, mouth ulcers, Cylindruria and hematuria in the SLE-MS group was statistically higher than that in the SLE-nMS group (all *P*<0.05) (Table-II).

**Table-II:** Comparison of clinical signs between SLE-MS group and SLE-nMS group [n(%)].

Variables		SLE-MS	SLE-nMS	χ^2^	P
Lupus nephritis	Yes	184(54.76)	334(37.03)	31.635	<0.001
	No	152(45.24)	568(62.97)		
Arthritis	Yes	122(36.3)	417(46.2)	9.802	0.002
	No	214(63.7)	485(53.8)		
Rash	Yes	167(49.7)	408(45.2)	1.966	0.161
	No	169(50.3)	494(54.8)		
Hair loss	Yes	33(9.8)	123(13.6)	3.235	0.072
	No	303(90.2)	779(86.4)		
Proteinuria	Yes	237 (70.5)	492(54.5)	25.854	<0.001
	No	99(29.5)	410(45.5)		
Oral ulcer	Yes	47(14.0)	87(9.6)	4.783	0.029
	No	289(86.0)	815(90.4)		
Fever	Yes	109(32.4)	331(36.7)	1.935	0.164
	No	227(67.6)	571(63.3)		
Thrombocytopenia	Yes	122(36.3)	282(31.3)	2.835	0.092
	No	214(63.7)	620(68.7)		
Leukopenia	Yes	239(71.1)	628(69.6)	0.265	0.607
	No	97(28.9)	274(30.4)		
Headache	Yes	13(3.9)	30(3.3)	0.215	0.643
	No	323(96.1)	872(96.7)		
Epilepsy	Yes	3(0.9)	3(0.3)	-	0.353 *
	No	333(99.1)	899(99.7)		
Brain syndrome	Yes	4(1.2)	5(0.6)	-	0.264 *
	No	332(98.8)	897(99.4)		
Mental illness	Yes	6(1.8)	10(1.1)	-	0.396 *
	No	330(98.2)	892(98.9)		
Optic neuritis	Yes	1(0.3)	0(0)	-	0.217 *
	No	335(99.7)	902(100)		
Cerebral neuritis	Yes	0(0)	5(0.56)	-	0.332 *
	No	336(100)	897(99.4)		
Cerebrovascular disease	Yes	1(0.3)	0(0)	-	0.271 *
	No	335(99.7)	902(100)		
Vasculitis	Yes	1(0.3)	1(0.1)	-	0.469 *
	No	335(99.7)	901(99.9)		
Tubular urine	Yes	37(11.0)	44(4.9)	15.063	<0.001
	No	299(89.0)	858(95.1)		
Hematuria	Yes	126(37.5)	251(27.8)	10.815	0.001
	No	210(62.5)	651(72.2)		

***Note:*** Significant results are highlighted in bold type.

* indicates the P value obtained by Fisher’s exact test.

Body mass index (BMI), SLE disease activity index (SLEDAI), systolic blood pressure (SBP), diastolic blood pressure (DBP), white blood cell count (WBC), mean red blood cell volume (MCV), red blood cell distribution width (RDW), urine microalbumin, urine red blood cell count (URBC), urinary tube type count (UTT), total protein (TP), triglyceride (TG), albumin (ALB), high density lipoprotein (HDL), globulin (GLB), low density lipoprotein (LDL), aspartate aminotransferase (AST), complement 3 (C3), urea (UA), creatinine (CREA), immunoglobulin A (IgA), fasting blood glucose (GLU), immunoglobulin G (IgG), total cholesterol (TC) and 24h urine protein (UP24) in SLE-MS group were statistically different from that in SLE-nMS group (all *P*<0.05) (Table-III).

**Table-III:** Comparison of Physical examination and laboratory parameters between SLE-MS group and SLE-nMS group.

Variables	SLE-MS	SLE-nMS	t /Z	P
Body mass index (BMI)	23.59±3.83	20.53±2.07	13.896	<0.001
SLE disease activity index (SLEDAI)	10 (7,13)	9 (5,13)	3.389	0.001
Systolic blood pressure (SBP)	138 (123,150)	120 (110,130)	12.888	<0.001
Diastolic blood pressure (DBP)	90 (80,100)	80 (70,86)	12.438	<0.001
White blood cell count (WBC)	5.32 (3.73,8.30)	4.80 (3.70, 7.10)	2.207	0.027
Red blood cell count (RBC)	3.87 (3.71,4.28)	3.97 (3.64, 4.25)	0.714	0.475
Neutrophil percentage	69.2 (63.1,77.85)	70.2 (61.0, 77.22)	0.175	0.861
Hemoglobin amount	113.0 (100.32,124.50)	114.0 (103.02,125.00)	1.043	0.297
Hematocrit	0.35 (0.32,0.39)	0.354 (0.324, 0.390)	0.689	0.491
Mean red blood cell volume (MCV)	88.5 (84.20,91.70)	85.70 (90.10, 94.80)	4.401	<0.001
Red blood cell distribution width (RDW)	14.25 (13.50,16.40)	14.10 (13.10,15.40)	2.455	0.014
Platelet count	137.75 (97.50,180.00)	140.0 (102.0,188.0)	0.351	0.726
Platelet distribution width (PDW)	16.40 (15.60,17.30)	15.30 (16.57,17.10)	0.469	0.639
Urine microalbumin	0.18 (0.15, 0.54)	0.15 (0.00, 0.26)	3.667	<0.001
Urine white blood cell count (UWBC)	17.30 (5.30,55.60)	13.60 (4.70,41.03)	1.423	0.155
Urine red blood cell count (URBC)	18.90 (5.90,67.60)	11.80 (4.70,37.05)	3.559	<0.001
Urinary tube type count (UTT)	0.64 (0.14,1.41)	0.51 (0.13,1.30)	2.103	0.035
Total protein (TP)	61.25 (53.10,68.10)	65.40 (60.00,71.00)	5.584	<0.001
Triglyceride (TG)	1.89 (1.33,2.68)	1.26 (0.93,1.61)	10.677	<0.001
Albumin (ALB)	33.50 (26.70,38.00)	35.60 (30.50,39.70)	4.242	<0.001
High density lipoprotein (HDL)	1.03 (0.83,1.35)	1.17 (0.90,1.46)	2.797	0.005
Globulin (GLB)	26.60 (22.20,32.40)	29.10 (25.10,34.00)	4.096	<0.001
Low density lipoprotein (LDL)	2.27 (1.71,3.17)	2.15 (1.63,2.80)	2.627	0.009
White ball ratio (WBR)	1.20 (0.90,1.50)	1.20 (1.00,1.40)	0.025	0.980
Aspartate aminotransferase (AST)	19.00 (12.00,32.25)	17.00 (11.00,29.00)	2.163	0.031
C-reactive protein (CRP)	5.91 (2.20,15.90)	4.72 (2.08,18.80)	0.537	0.591
Alanine aminotransferase (ALT)	22.00 (16.00,32.00)	21.00 (17.00,31.00)	0.062	0.951
Complement 3 (C3)	0.82 (0.55,1.06)	0.73 (0.54,0.95)	2.000	0.045
Urea (UA)	6.96 (4.68,11.74)	4.78 (3.68,6.57)	7.883	<0.001
Complement 4 (C4)	0.14 (0.06,0.24)	0.14 (0.07,0.22)	0.248	0.804
Creatinine (CREA)	72.60 (53.65,136.88)	59.60 (48.55,74.98)	6.029	<0.001
Immunoglobulin A (IgA)	2.43 (1.71,3.31)	2.63 (2.01,3.54)	2.258	0.024
Fasting blood glucose (GLU)	5.85±2.50	4.91±1.69	5.857	<0.001
Immunoglobulin G (IgG)	12.60 (9.42,17.60)	15.30 (11.57,19.40)	4.585	<0.001
Total cholesterol (TC)	4.59 (3.44,5.85)	3.97 (3.23,4.85)	5.153	<0.001
Immunoglobulin M (IgM)	0.91 (0.50,1.34)	0.96 (0.60,1.44)	1.437	0.151
Immunoglobulin E (IgE)	57.88 (18.25,212.22)	56.70 (16.46,189.75)	0.733	0.464
Erythrocyte sedimentation rate (ESR)	40.40 (19.85,72.00)	37.05 (17.00,67.40)	1.598	0.110
24h urine protein (UP24)	1.14 (0.29,2.44)	0.34 (0.12,1.38)	4.777	<0.001

***Note:*** Significant results are highlighted in bold type.

**Fig.1:**
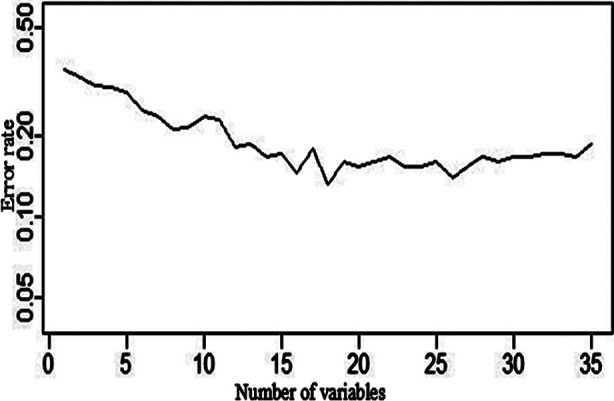
Classification error rate graph by the SWSFS process. ***Abbreviations:*** **SWSFS:** sliding window sequential forward feature selection.

We established a random forest model with or without MS as the dependent variable, and the statistically significant index after the univariate analysis as the independent variable. The results showed that the top 18 variables ranked by importance score were: BMI, SBP, DBP, Glu, UP, arthritis, occupation, UA, TG, education, HDL, WBC, IgG, C3, LDL, MCV, SLEDAI, GLB (Table-IV). We ran the SWSFS process and plotted the error rate graph. The results showed that the error rate is the lowest when the number of independent variables is 18 (Fig.1).

**Table-IV:** Variable importance score by random forest algorithm.

Variables	Importance score
BMI	10.28
DBP	9.39
SBP	7.39
GLU	4.31
UP	3.41
Arthritis	2.93
Occupation	2.22
UA	1.59
TG	1.59
Education	1.58
HDL	1.56
WBC	1.45
IgG	1.38
C3	1.28
LDL	1.22
MCV	1.18
SLEDAI	1.17
GLB	1.06
IgA	1.05
CREA	1.03
LN	1.01
UP24	0.94
Mouthulcer	0.93
ALT	0.93
RDW	0.89
ALB	0.80
TP	0.78
Proteinuria	0.72
Hematuria	0.71
TC	0.69
URBC	0.68
Urine microalbumin	0.58
UTT	0.52
Cylindruria	0.48
Marriage	0.46

***Abbreviations:*** BMI: body mass index; DBP: diastolic blood pressure; SBP: systolic blood pressure; GLU: fasting blood glucose; UP: Urinary protein; UA: urea; TG: triglyceride;

HDL: high density lipoprotein; WBC: white blood cell count; IgG: immunoglobulin G;

C3: complement 3; LDL: low density lipoprotein; MCV: mean red blood cell volume;

SLEDAI: systemic lupus erythematosus disease activity index; GLB: globulin; IgA: immunoglobulin A;

CREA: creatinine; LN: lupus nephritis; UP24: 24h urine protein.

We performed binary logistic regression analysis with or without MS as the dependent variable and the top 18 indicators of variable importance score as independent variables. The results showed that BMI, DBP, SBP, GLU, arthritis, UA, TG, and HDL were associated with MS in SLE (Table-V).

**Table-V:** Binary logistic regression analysis of influencing factors regarding MS in SLE.

Independent variable	β	s_x_	Waldχ^2^	P	OR (95%CI)
Body mass index (BMI)	0.443	0.079	31.408	<0.001	1.557 (1.334, 1.818)
Diastolic blood pressure (DBP)	0.07	0.017	16.233	<0.001	1.073 (1.037,1.110)
Systolic blood pressure (SBP)	0.031	0.012	7.014	0.008	1.031 (1.008,1.055)
Fasting blood glucose (GLU)	1.338	0.208	41.478	<0.001	3.813 (2.537,5.730)
Arthritis	1.385	0.389	12.656	<0.001	3.995 (1.863,8.569)
Urea (UA)	0.071	0.03	5.444	0.020	1.074 (1.011,1.139)
Triglyceride (TG)	0.86	0.18	22.904	<0.001	2.364 (1.662,3.362)
High density lipoprotein (HDL)	-1.812	0.504	12.929	<0.001	0.163 (0.061,0.439)
White blood cell count (WBC)	0.09	0.052	3.709	0.079	1.095 (0.989,1.211)
Constant	-29.406	3.385	75.455	<0.001	<0.001

***Note:*** Significant results are highlighted in bold type.

## DISCUSSIONS

The clinical manifestations of almost all autoimmune diseases are likely to occur in SLE. Therefore, most scholars call it the prototype of autoimmune diseases, and its pathogenesis is still unclear.^15^ MS is related to the occurrence of CVD in SLE patients. Preventing the occurrence of MS in SLE patients is beneficial to the control of their condition. This study showed that the prevalence of MS in SLE patients is 27.14%, which is lower than the results of scholars in South America and Europe,^16^,^17^ which may be related to the different samples from different regions and the diagnostic criteria used. The results of our previous survey showed that the MS prevalence rate of community physical examination population was 13.4%,^18^ so the MS prevalence rate of SLE patients was significantly higher than that of community physical examination population. SLE is an important public health problem in the world. MS is an independent risk factor for CVD in SLE. Exploring the related factors of MS in SLE patients is of great significance for controlling the occurrence and development of SLE.

This study showed that MS in SLE patients is associated with farmers, married, and low levels of education. This may be because farmers have a relatively low level of education, low economic income, poor self-care awareness, and less access to health knowledge. Haiyan Shi et al. found that the middle-aged population generally lacks knowledge of MS and has not even heard of MS.^19^ Therefore, the prevention and treatment of MS in SLE patients should focus on farmers and low-education populations, and their health knowledge promotion and health services should be strengthened.

Lupus nephritis (LN) is a serious and common complication of SLE, which can significantly increase the incidence and mortality of SLE.^20^ This study found that the occurrence of MS in SLE patients is related to LN, which is consistent with the results of Zhang M et al.^21^ LN patients have a variety of autoimmune lymphocytes and autoantibodies, which can activate endothelial cells and cause CVD. Therefore, LN itself is a risk factor for CVD in SLE patients. The risk factors of LN include family history, smoking history, age, etc. At the same time, the components of blood glucose, blood pressure, blood lipids and body weight in MS interact with each other in metabolism, which is also an important risk factor for LN. Moreover, the proportion of arthritis, proteinuria, mouth ulcer, tubular urine and hematuria in SLE-MS patients was significantly different from that in SLE-nMS group (*P*<0.05), suggesting that symptomatic treatment of these signs in clinical practice is of great significance for the prevention and control of MS in SLE.

Red blood cell distribution width (RDW) is a parameter that directly describes red blood cell heterogeneity and is generally used to differentially diagnose thalassemia, giant cell anemia, and iron deficiency anemia. Recently, RDW has been found to be a strong independent predictor of all-cause mortality in coronary atherosclerotic heart disease (CAD), acute coronary syndrome (ACS), hypertension, and diabetic nephropathy.^22^,^23^ Univariate analysis of this study showed that RDW was associated with SLE-MS, similar to our previous survey of metabolic syndrome in community physical examination populations, suggesting that RDW, a simple, inexpensive and readily available parameter, has good early warning and prognostic evaluation values in SLE-MS.^18^ The univariate analysis of this study showed that SLEDAI was associated with MS in SLE, indicating that MS may affect the severity of SLE disease.

Multivariate logistic regression analysis of this study showed that BMI, SBP, DBP, GLU, TG, and HDL were related to MS in SLE. These reflect the relationship between obesity, hypertension, hyperglycemia, dyslipidemia and MS in the general population also exists in SLE patients.^24^,^25^ Multivariate analysis showed that arthritis was a risk factor for MS in SLE. Lee et al. found that the prevalence of MS in healthy controls was significantly lower than in patients with rheumatoid arthritis and the results support the views of this paper.^26^

### Limitations of study

Several limitations exist in the current study. The lifestyle and dietary habits of the subjects in this study were not analyzed, so they need to be improved in future work. The patients with SLE in this study were recruited from only one hospital and the promotion of conclusions needs to consider regional factors. In addition, distorted results in epidemiological association studies can be resulted in by potential biases in case–control studies. Consequently, further studies are needed to clarify the exact influencing factors on MS in SLE.

## CONCLUSION

The occurrence of MS in SLE patients is closely related to BMI, blood pressure, blood sugar, blood lipids, and arthritis.

### Authors’ contributions:

**HY** designed the study and conceived the survey, evaluated the results and revised the manuscript.

**JZ and MJT** collected epidemiological data.

**LX and HP** sorted the data.

**QL and ZL** were involved in the fieldwork.

**LRJ** conducted the analysis and wrote the first draft of the manuscript.

All authors approved the final version of the manuscript and contributed toward data analysis, drafting and revising the paper and agree to be accountable for all aspects of the work.
